# Acupuncture for type 2 diabetes mellitus with nonalcoholic fatty liver disease: A protocol for systematic review and meta-analysis

**DOI:** 10.1097/MD.0000000000026043

**Published:** 2021-05-28

**Authors:** Mengyuan Li, Lin Yao, Haipeng Huang, Guan Wang, Bin Yu, Haizhu Zheng, Hongfeng Wang

**Affiliations:** Changchun University of Chinese Medicine, Nanguan District, Changchun, Jilin, China.

**Keywords:** acupuncture, meta-analysis, nonalcoholic fatty liver disease, systematic review, type 2 diabetes mellitus

## Abstract

**Background:**

T2DM with NAFLD is a common disorder of glucose and lipid metabolism affecting the quality of life of patients. Due to the limitations and adverse reactions of drug treatment, acupuncture has been proved to be an effective method for the treatment of T2DM with NAFLD. This study aims to evaluate the efficacy and safety of acupuncture in the treatment of NAFLD in T2DM class, and to provide high-quality evidence for acupuncture in the treatment of this disease.

**Methods:**

From establishment of the database to 31 July 2021, We will search the Cochrane Central Register of controlled trials, PubMed, MEDLINE, EMBASE, Web of science. Five Chinese databases, including China National Knowledge Infrastructure (CNKI), WanFang database, VIP, Chinese Biomedical Literature Database (CBM) and the Chinese clinical trial registry. There are no restrictions on language or publication, and they are independently screened and collected by two reviewers. Review Manager 5.3 software will be used for meta analysis. If necessary, heterogeneity testing, data synthesis, and subgroup analysis will be performed.

**Results:**

The effectiveness and safety of acupuncture in the treatment of T2DM with NAFLD will be assessed by the outcomes of test's, including: imaging indicators, biomarkers of hepatic steatosis, serological indicators of hepatic fibrosis, improvement of serum NAFLD liver fat score, BMI, blood glucose indexes, blood lipid indexes, insulin level and safety indicators.

**Conclusion:**

This meta-analysis will further determine the beneficial efficacy and safety of acupuncture for T2DM combined with NAFLD.

## 1 Introduction

Diabetes mellitus (DM) is one of the most common chronic metabolic diseases and the increasing global prevalence and mortality of DM makes it an important topic of medical research.^[1]^ According to the International Diabetes Federation (IDF), there are approximately 463 million people with diabetes worldwide in 2019, representing 9.3% of the global population, 80% of them are found in underdeveloped countries.^[2]^ Type 2 diabetes mellitus (T2DM) is far more usual than type 1 diabetes or gestational diabetes, approximately 90% of DM.^[3,4]^ Nonalcoholic fatty liver disease (NAFLD) has been indicated to be closely linked to T2DM as one of the most common liver disorders worldwide.^[5]^ Growing evidence suggests that the prevalence of NAFLD in T2DM has substantially increased.^[6]^ The combination of T2DM and NAFLD often causes adverse consequences, which not only increases the risk of diabetic complications, but also is a vital factor for liver cirrhosis and liver cancer.^[7]^ Studies have shown that the relationship between T2DM and NAFLD is mutual and bidirectional. NAFLD can accelerate the development of T2DM, but the improvement of NAFLD can also reduce the risk of T2DM.^[8]^ Therefore, the prevention and treatment of T2DM with NAFLD have become the key issues to be solved in modern medicine.

The pathogenesis associated with T2DM with NAFLD includes insulin resistance (IR), oxidative stress imbalance, free fatty acid accumulation, and endoplasmic reticulum stress.^[9–12]^ Nevertheless, the potential mechanism of T2DM with NAFLD remains to be clarified. Currently, several studies have shown that IR is an important cause of the development of T2DM with NAFLD.^[13,14]^ In the development, progression, and determination of the severity of pathogenesis of T2DM with NAFLD, IR plays a key role. One of the most convincing evidence is that several modified NAFLD mice, including SREBP-1c transgenic mice, ob/ob, and db/db mice, can induce insulin resistance.^[15,16]^ Therefore, modulation of IR is a potential strategy for NAFLD treatment. At present, the treatment of T2DM with NAFLD in modern medicine is mainly based on strict control of blood glucose and diet, giving Metformin, Nitrotyrosine, α-glucosidase inhibitors, Glucagon-like peptide 1 receptor (GLP-1) agonists, Dipeptidyl peptidase 4 (DPP-4) inhibitors, Peroxisome proliferator-activated receptor (PPAR-γ/α/δ) agonist.^[17–20]^ The mechanisms of these drugs are primarily used to improve IR. However, long-term application is prone to adverse reactions, and it also causes great economic burden to patients.

Acupuncture, as an in vitro treatment of traditional Chinese medicine, has played a huge advantage in regulating diseases such as glycolipid metabolism disorders. A large number of studies have shown that acupuncture can significantly improve dyslipidemia in diabetic patients, and the curative effect is better than drug therapy.^[21,22]^ Acupuncture can not only regulate fat accumulation in the liver of obese people,^[23]^ reduce cholesterol levels, regulate the expression of different genes in the liver,^[24]^ but also regulate the expression of decoupling proteins in skeletal muscle, make white adipose tissue into brown adipose tissue, and promote weight loss.^[25]^ The above studies show that acupuncture has a benign regulatory effect on glycolipid metabolism disorders, and it is safe, less side effects, and easy to be accepted by patients. Its mechanism involves multiple signaling pathways and multiple targets.

However, there are few studies on the effect of acupuncture on T2DM with NAFLD, and the research quality is uneven. Moreover, there is no systematic review and meta-analysis on the treatment of this disease by acupuncture. Thus, on the basis of evidence-based medicine, we will collect randomized controlled trials (RCTs) of acupuncture in the treatment of T2DM with NAFLD, and conduct a meta-analysis of its efficacy and safety, providing higher quality clinical evidence for acupuncture in the treatment of NAFLD in diabetic patients.

## 2 Methods

The protocol follows the Cochrane Handbook for Preferred Reporting Items for Systematic Reviews and Meta-analyses Protocols (PRISMA-P) 2015.^[26,27]^ It was registered on INPLASY202140140. If needed, we will describe the modifications in our full review.

### 2.1 Eligible criteria for selection of studies

#### 2.1.1 Types of studies

We will only include RCTs that are more likely than other study designs to provide impartial details. RCTs assessing the efficacy of acupuncture in the treatment of T2DM with NAFLD will include, whether blinded or not. The following types of articles will be excluded: case reports, observational studies, retrospective studies, animal experiments, and review articles. No restrictions on language and publication time.

#### 2.1.2 Types of participants

We will include patients no matter sex (over 18 years old) who have been diagnosed T2DM with NAFLD. The diagnosis of diabetes must be based on the medical diagnostic criteria for diabetes released by the American Diabetes Association (ADA) in 2019, ^[28]^ while meeting the compatibility of NAFLD ultrasound and hepatic steatosis. Body mass index between 25 and 40. The age, sex, or ethnicity of the enrolled subjects is not limited.

#### 2.1.3 Types of interventions

Experimental interventions: the main intervention methods include manual acupuncture, electroacupuncture, moxibustion, ear acupuncture, scalp acupuncture, regardless of acupuncture techniques, and stimulation methods. Studies in which therapy without needling, such as acupressure, tap pricking, point injection, and laser acupuncture, will be excluded.

Comparator interventions: The main intervention methods are drugs, placebo, sham acupuncture or no treatment. Sham acupuncture refers to the stimulation of nonacupoints.

We will investigate the following comparisons:

1. Acupuncture compared with placebo;
2. Acupuncture compared with sham acupuncture;
3. Acupuncture compared with drugs;
4. Acupuncture compared with no treatment.

### 2.2 Types of outcome measures

#### 2.2.1 Primary outcomes

Imaging indicators, biomarkers of hepatic steatosis, serological indicators of hepatic fibrosis, improvement of serum NAFLD liver fat score, and clinical efficacy.

#### 2.2.2 Secondary outcomes

1. Body mass index;
2. Blood glucose indexes: fasting blood glucose, 2 hours blood glucose after breakfast, glycosylated hemoglobin;
3. Blood lipid indexes: total cholesterol (TC), high-density lipoprotein cholesterol, triglyceride, and low-density lipoprotein cholesterol (LDL-C);
4. Insulin level: fasting and 2 hours postprandial blood glucose;
5. Safety indicators: alanine aminotransferase, creatinine, aspartate aminotransferase, urea nitrogen, hemoglobin, white blood cell count (WBC), and platelet count.

### 2.3 Searching methods

#### 2.3.1 Electronic searches

We will search the Cochrane Central Register of controlled trials, PubMed, MEDLINE, EMBASE, Web of science. Five Chinese databases, including China National Knowledge Infrastructure (CNKI), WanFang database, VIP, Chinese Biomedical Literature Database (CBM), and the Chinese clinical trial registry. We will use free combination of keywords and subject words. The search term will include: “acupuncture,” “manual acupuncture,” “electroacupuncture,” “moxibustion,” “ear acupuncture,” “scalp acupuncture,” “acupoints,” “type 2 diabetes mellitus,” “type 2 diabetes,” “diabetes,” “diabetes mellitus,” “nonalcoholic fatty liver disease,” “nonalcoholic fatty liver,” “nonalcoholic steatohepatitis”, “NAFLD”, “fatty liver”, “liver fibrosis”, “liver cirrhosis.” The Chinese translation of these search terms will be used in Chinese databases. There will be no language limits and the last retrieval date on July31, 2021. The search strategy for Pubmed is shown in Table 1. Adjust search strategy according to different database requirements.

**Table 1:** Search strategy in Pubmed (MEDLINE).

Number	Search terms
#1	Type 2 diabetes mellitus (MeSH)
#2	Type 2 diabetes (MeSH)
#3	Diabetes mellitus (MeSH)
#4	Diabetes (MeSH)
#5	T2DM (MeSH)
#6	Or #1–#5
#7	Nonalcoholic fatty liver disease (MeSH)
#8	Nonalcoholic fatty liver (MeSH)
#9	Nonalcoholic steatohepatitis (MeSH)
#10	Fatty liver (MeSH)
#11	Liver fibrosis (MeSH)
#12	Liver cirrhosis (MeSH)
#13	NAFLD
#14	Or #7–#13
#15	Acupuncture (MeSH)
#16	Manual acupuncture (MeSH)
#17	Electroacupuncture.ab.
#18	Moxibustion.ab.
#19	Ear acupuncture
#20	Scalp acupuncture
#21	Acupoints (MeSH)
#22	Or #15–#21
#23	Randomised controlled trial.pt.
#24	Controlled clinical trial.pt.
#25	Clinical Trial.pt.
#26	Placebo.ab.
#27	Clinical trials as topic (MeSH)
#28	Pragmatic clinical trial.pt.
#29	Or #23–#28
#30	#6 AND #14 AND #22 AND #29
#31	Remove duplicates from #30

#### 2.3.2 Search other resources

At the same time, we will use other search resources to supplement electronic databases. The WHO International Clinical Trials Registry Platform (https://apps.who.int/trialsearch), the US National Institutes of Health Ongoing Trials Register (https://clinicaltrials.gov/ct2/home) will be searched for any relevant ongoing or unpublished trials. Google Scholar (https://scholar.google.com) will be also used for searching string “acupuncture” AND “T2DM” AND “NAFLD” for any possible related trials.

### 2.4 Data collection and analysis

#### 2.4.1 Selection of studies

**Figure 1:**
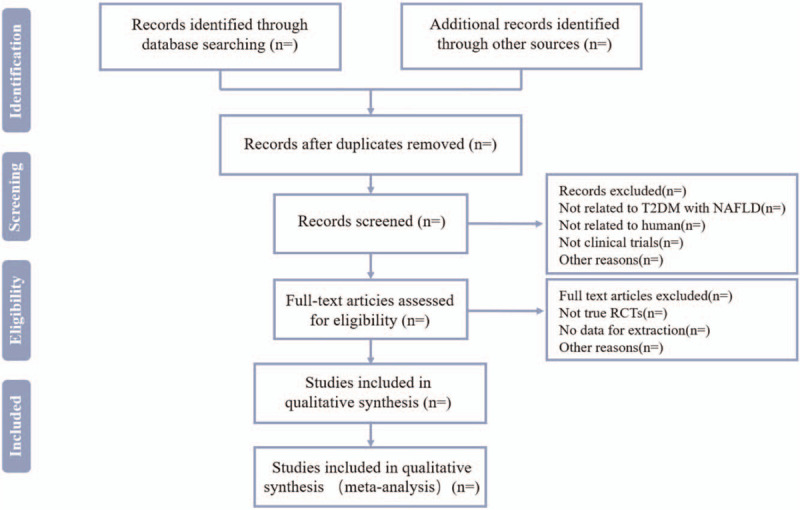
Flow diagram of the study selection process.

First, 2 review authors will independently search the literature to determine the literature titles and abstracts. Then, the identified literature will import into EndnoteX9 software to remove duplicates. Second, the identified literature will be downloaded in full text, read carefully and relevant works against the predefined inclusion criteria will be selected to further decide whether to include it or not. Finally, the reviewers cross-check the inclusion results. If there are differences in the process of verification, we will seek the opinions of the third party. PRISMA diagram (Fig. 1) will be used for illustrating the selection process.

### 2.5 Data extraction and management

We will prepare a data template designed in advance, including the following information: baseline information, study population, research characteristics, intervention methods, primary and secondary outcomes collection, adverse events and follow-up date. Data collection for this template will be finished by 2 researchers independently. Finally, the data collected by the 2 researchers will be checked. It will be discussed by reviewers, if there is any difference. For studies with missing data or unclear information, we will attempt to contact the original authors to obtain accurate data. Any difference will be resolved through negotiation or by seeking third-party advice.

### 2.6 Assessing risk of bias in included studies

Using the Cochrane Review Handbook 5.2 recommended quality evaluation criteria - bias risk assessment tool. It mainly involves 7 aspects:

1. random assignment method;
2. allocation protocol concealment;
3. blinding of study subjects and treatment protocol implementers (assessed for each study outcome);
4. blinding of outcome measures (assessed for each study outcome);
5. completeness of outcome data (assessed for each study outcome);
6. selective reporting of study results; and
7. other sources of bias. For each included study, the above 7 aspects were judged, including 3 criteria: “low risk,” “high risk,” and “unclear.”

Two researchers will summarize the results of the quality evaluation of the included trials separately, and a third party will assist in resolving those that disagree or are difficult to determine through unclear discussion.

### 2.7 Grading the quality of evidence

We will use the Grading of Quantitative Systematic Assessment of Evidence (GRADE) tool to evaluate the quality of the literature, classifying the quality of evidence into “high, medium, low and very low” and the strength of recommendation into “strong recommendation and weak recommendation.”

### 2.8 Measures of treatment effect

For continuous variable results, we will report the mean difference (MD) or standardized mean difference (SMD) and the 95% confidence interval (CI). We will report the relative risk (RR) and 95% CI for dichotomous outcomes.

If MD or SMD were not reported, we recalculated SD and SMD using the original data, such as medians and confidence intervals, based on relevant information recorded in the study.

### 2.9 Data synthesis and analysis

Meta-analysis will be performed using the software RevMan 5.3 (where the publication bias section will be performed using stata software). Heterogeneity tests were performed among studies, using *I*^2^ as an evaluation index, and *I*^2^ ≤ 50% and *P* > .1, heterogeneity was considered small and a fixed-effect model should be selected. *I*^2^ = 50%, *P* < .1, the heterogeneity is large. The random effect model should be selected, and sensitivity analysis or subgroup analysis should be carried out to explore the source of heterogeneity. Wighted mean difference (WMD) was used for continuous variables; RR was used for categorical variables, and each effect size was expressed with 95% CI, and differences were considered statistically significant at *P* ≤ .05.

### 2.10 Dealing with missing data

If the data needed in the meta-analysis is incomplete, the reviewer will attempt to contact the first author or corresponding author by phone or email to obtain the missing data. If missing data cannot be obtained by these means, a sensitivity analysis will be used to evaluate the potential effect on the overall results of the study of the missing data.

### 2.11 Subgroup analysis

If data are sufficient, we will investigate heterogeneity by grouping analyses according to age, gender, acupuncture intervention (acupuncture, electroacupuncture), different control group types (placebo, sham acupuncture, no treatment or medication), duration of treatment, and duration of symptoms at baseline, and duration of treatment sessions.

### 2.12 Sensitivity analysis

Sensitivity analysis is an important method used in meta-analysis to assess the robustness and reliability of the combined results. The specific methods are as follows:

1. Changing the analysis model: when heterogeneity is high (*I*^2^ > 50%), a random-effects model is recommended, and conversely, a fixed-effects model is used.
2. Excluding literature one by one: exclude each included study one by one before combining effect sizes, change the inclusion exclusion criteria or exclude certain types of literature before combining effect sizes.

### 2.13 Ethics and dissemination

This meta-analysis and analysis results will be published in peer review journals and published in relevant conferences. Without ethical approval, the data we use do not contain personal data of patients and do not need to worry about patients’ privacy. This review will comprehensively evaluate the effect of acupuncture and moxibustion on T2DM with NAFLD.

## 3 Discussion

The prevalence of metabolic diseases, mainly T2DM, is increasing globally every year, and more than four fifths of patients are found in developing countries, posing a serious physical and economic burden to human beings. NAFLD is one of the most common liver diseases and an important component of metabolic diseases, the incidence of which is also increasing every year. NAFLD is an independent risk factor for increased cardiovascular and cerebrovascular events in T2DM patients. Similarly, the presence of T2DM increases the risk of inflammatory changes and even fibrosis in hepatocytes. The 2 have been shown to influence each other and to be risk factors for each other's progression.^[29]^

A large number of studies have shown that acupuncture can significantly improve dyslipidemia in diabetic patients and is no less effective than drug therapy. From the current state of research, acupuncture has a benign regulatory effect on lipid metabolism, and its mechanism of action involves multiple signaling pathways and multiple targets. It should be noted that acupuncture and NAFLD are also associated with IR and T2DM. It is certain that NAFLD is closely related to T2DM. However, there are still the following problems. Firstly, there are few studies on the mechanism of effect of acupuncture on liver lipid accumulation in the diabetic state. Secondly, there is a lack of convincing evidence-based medicine to prove the efficacy of acupuncture in the treatment of T2DM with NAFLD. Therefore, we conducted this meta-analysis to analyze and summarize the efficacy and safety of acupuncture in the treatment of T2DM with NAFLD. This meta-analysis provides a comprehensive assessment of whether acupuncture is effective in T2DM with NAFLD. And the findings will provide additional evidence for the therapeutic effect of acupuncture on T2DM with NAFLD.

In summary, we hope that this systematic review and meta-analysis will provide high-quality evidence for the efficacy of complementary acupuncture in T2DM with NAFLD, and have a beneficial impact on patients.

## Author contributions

**Conceptualization:** Lin Yao, Guan Wang, Hongfeng Wang.

**Data curation:** Haizhu Zheng.

**Formal analysis:** Lin Yao.

**Funding acquisition:** Hongfeng Wang.

**Investigation:** Guan Wang.

**Methodology:** Mengyuan Li, Haizhu Zheng.

**Project administration:** Bin Yu.

**Resources:** Bin Yu.

**Supervision:** Haipeng Huang.

**Visualization:** Mengyuan Li.

**Writing – original draft:** Mengyuan Li.

**Writing – review & editing:** Haipeng Huang, Hongfeng Wang.
